# Supplementary material for: Gentle Touch Therapy, Pain Relief and Neuroplasticity at Baseline in Fibromyalgia Syndrome: A Randomized, Multicenter Trial with Six-Month Follow-Up
Source: J Clin Med. 2022 Aug 20;11(16):4898. doi: 10.3390/jcm11164898 (PMC9410244; doi:10.3390/jcm11164898)
Supplement: Supplementary file 1 [file jcm-11-04898-s001.zip › jcm-1770806-supplementary.pdf]

Supplementary Material

Supplementary Figure S1A: Example of treatment direction with gentle touch therapy using both hands.

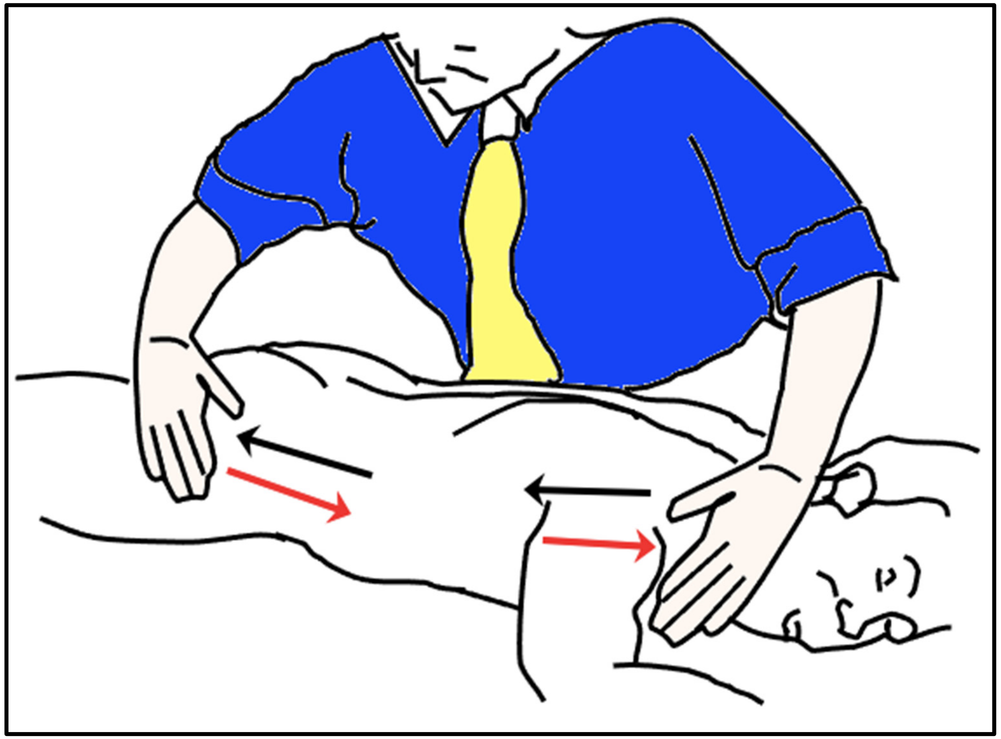

Adapted from Salgado (2019).

Salgado, A. S. I. Fisioterapia Integrativa: como ter saúde em um mundo doente. Midiograf: Londrina, Brasil, 2019; p 304.

Supplementary Figure S1B: Main regions of dermal projections of muscles related to their metameric of origin (muscle embryonic correspondence in the dermis).

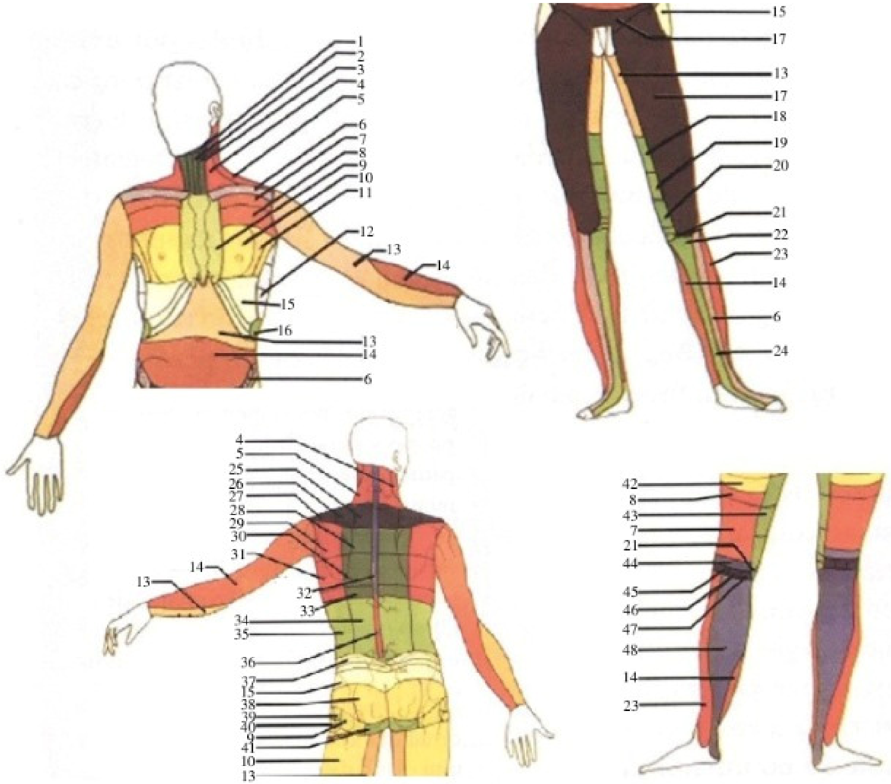

1 vastus intermedius m.
2 vastus medials m.
3 vastus lateralis m.
4 biceps femoris long head m.
5 biceps femoris short head m.
6 central band
7 sternocleidomastoid m.
8 orbicularis oculi m. (palpebral part)
9 interpharyngeal m.
10 facial expression m. (inferior)
11 facial expression m. (superficial)
12 eyeball motor muscles (superior, inferior and medium)
13 upper band
14 medium band
15 facial expression muscle (superior)
16 gluteus maximus muscle (profound)
17 lower band
18 thoracic diaphgragm m. (anterior)
19 thoracic diaphgragm m. (medium)
20 thoracic diaphgragm m. (inferior)
21 pelvic floor m.
22 intercostal m. (T1)
23 longitudinal m. (C1-L5)
24 intercostal m. (T8)
25 semimembranosus m. (reflex)
26 semimembranosus m. (direct)
27 semimembranosus m. (recurrent)
28 piriformis m.
29 gluteus medius and minimus m.
30 quadratus femoris m.
31 obturator internus and externus m.
32 rectus cpitat posterior m. (larger)
33 gluteus maximus m. (coccygeal)
34 gluteus maximus m. (superior)
35 gluteus maximus m. (medium)
36 oblique larger of the head m.
37 occipital m.
38 facial expression m. (medium)
39 stapedius m.
40 tensor tympani m.
41 tongue floor m.
42 plastysma m.
43 thoracic diaphgragm m.
44 axial m. (C1)
45 longitudinal m. (neck)
46 rectus cpitat anterior m. (larger)
47 rectus cpitat anterior m. (smaller)
48 axial m. (C2-L5)

Legend: m. = muscle.

Adapted from Grosjean (2016).

Grosjean, D. Investigação da Etiologia em Microfisioterapia.: Andreoli: São Paulo, Brasil, 2016; pp 160.

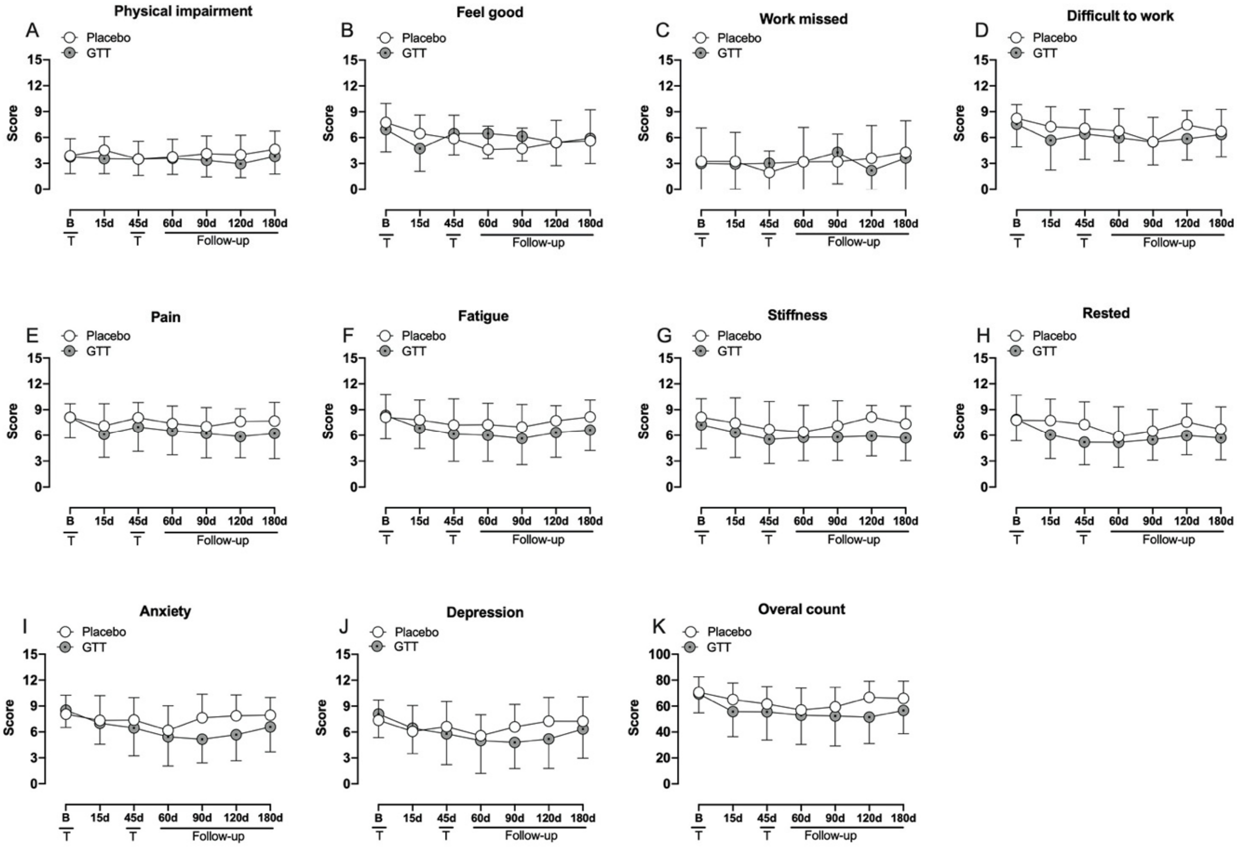

Supplementary Figure S2. Gentle touch therapy and Fibromyalgia Impact Questionnaire (FIQ). Panels A-K shows the evaluations at different times. Repeated-measures two-way analysis of variance followed by Bonferroni post hoc test. GTT: Gentle touch therapy group; d: day.

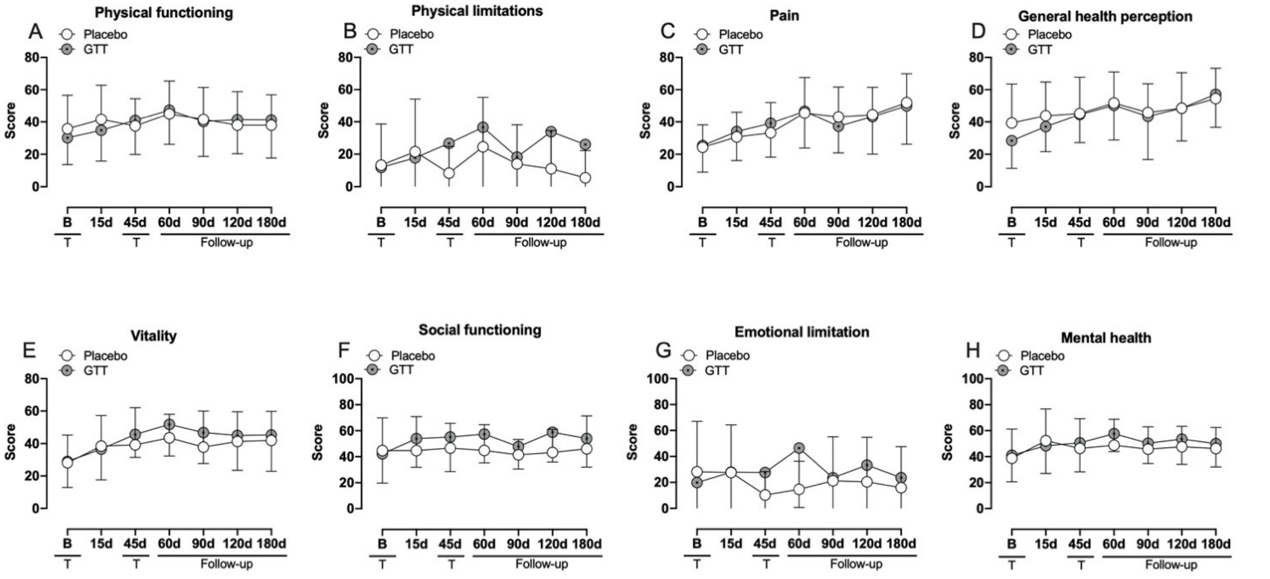

Supplementary Figure S3. Gentle touch therapy and Short-Form Health Survey (SF-36). Panels A-H shows the evaluations at different times. Repeated-measures two-way analysis of variance followed by Bonferroni post hoc test. GTT: Gentle touch therapy group; d: day.

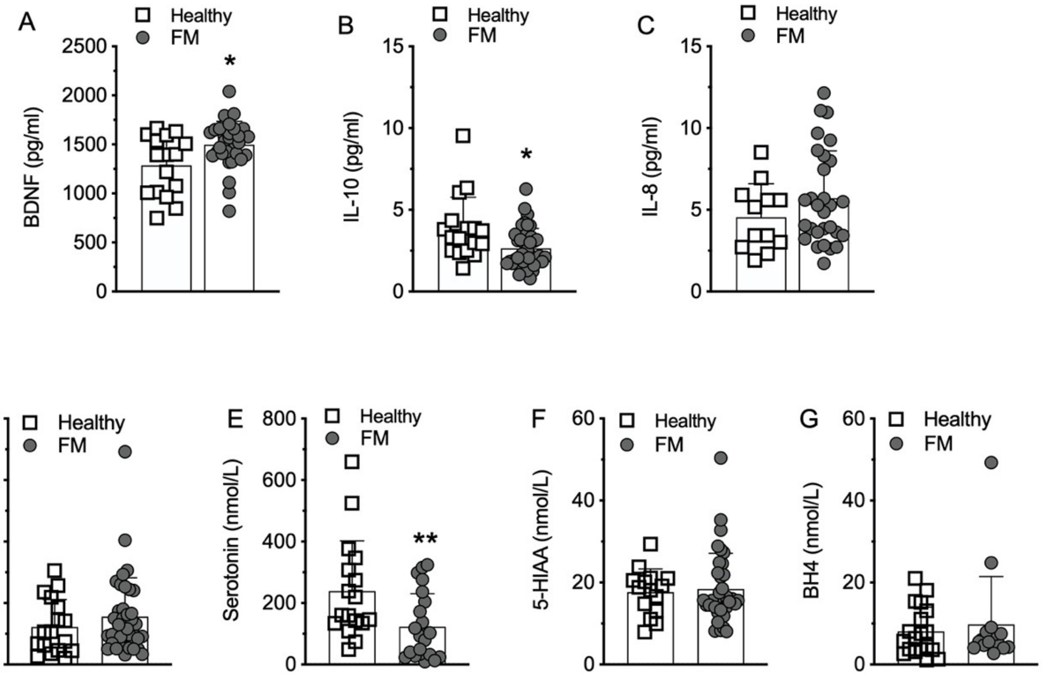

Supplementary Figure S4. Serum levels of brain-derived neurotrophic factor (BDNF), interleukin-10 (panel B), interleukin-8 (panel C), dopamine (panel D), serotonin (panel E), 5-hydroxyindolacetic acid (5-HIAA, panel F) and urinary level of tetrahydrobiopterin (BH4, panel G) comparing FM vs healthy individual groups. Unpaired Student t-test was used for the parametric data or the Mann-Whitney test for the non-parametric data. $^{*}p < 0.05$ and $^{**}p < 0.01$. FM: Individual with fibromyalgia group; Healthy: healthy individual group; Before: baseline; After: after 60 days.
